# Medium Energy Carbon and Nitrogen Ion Beam Induced Modifications in Charge Transport, Structural and Optical Properties of Ni/Pd/n-GaN Schottky Barrier Diodes

**DOI:** 10.3390/ma13061299

**Published:** 2020-03-13

**Authors:** Santosh Kumar, Xiang Zhang, Vinay Kumar Mariswamy, Varra Rajagopal Reddy, Asokan Kandasami, Arun Nimmala, S V S Nageswara Rao, Jue Tang, Seeram Ramakrishnna, Krishnaveni Sannathammegowda

**Affiliations:** 1 School of Mechanical Engineering, Beijing Institute of Technology, Beijing 100081, China; santosh@physics.uni-mysore.ac.in; 2 Department of Studies in Physics, Manasagangotri, University of Mysore, Mysuru 570006, India; 3 Department of Physics, K.L.E Society’s R.L.S Institute, Belagavi 590001, India; vkm288@gmail.com; 4 Department of Physics, Sri Venkateswara University, Tirupati 517502, India; reddy_vrg@rediffmail.com; 5 Material Science Group, Inter-University Accelerator Centre (IUAC), New Delhi 110067, India; asokan@iuac.res.in; 6 Centre for Advanced Studies in Electronics Science and Technology (CASEST), School of Physics, University of Hyderabad, Hyderabad 500046, India; arun.nimmala07@gmail.com (A.N.); svnsp@uohyd.ac.in (S.V.S.N.R.); 7 School of Physics, University of Hyderabad, Hyderabad 500046, India; 8 Business School, Guilin University of Technology, Guilin 541004, Guangxi, China; 9 Department of Mechanical Engineering, National University of Singapore, Singapore 117576, Singapore; seeram@nus.edu.sg

**Keywords:** GaN Schottky Diodes, MEI irradiation, NIEL, LET, Electrical parameters, Charge transport mechanism, surface morphology, optically active defects

## Abstract

The irradiation effects of carbon and nitrogen medium energy ions (MEI) on charge transport, structural and optical properties of Ni/Pd/n-GaN Schottky barrier diodes are reported. The devices are exposed to 600 keV C^2+^ and 650 keV N^2+^ ions in the fluence range of 1 × 10^13^ to 1 × 10^15^ ions cm^−2^. The SRIM/TRIM simulations provide quantitative estimations of damage created along the trajectories of ion beams in the device profile. The electrical parameters like Schottky barrier height, series resistance of the Ni/Pd/n-GaN Schottky barrier diodes decreases for a fluence of 1 × 10^13^ ions cm^−2^ and thereafter increases with an increase in fluence of 600 keV C^2+^ and 650 keV N^2+^ ions. The charge transport mechanism is influenced by various current transport mechanisms along with thermionic emission. Photoluminescence studies have demonstrated the presence of yellow luminescence in the pristine samples. It disappears at higher fluences due to the possible occupancy of Ga vacancies. The presence of the green luminescence band may be attributed to the dislocation caused by the combination of gallium vacancy clusters and impurities due to MEI irradiation. Furthermore, X-ray diffraction studies reveal that there is a decrease in the intensity and shift in the diffraction peaks towards the lower side of two thetas. The reductions in the intensity of C^2+^ ion irradiation is more when compared to N^2+^ ion irradiation, which may be attributed to change in the mean atomic scattering factor on a given site for light C^2+^ ion as compared to N^2+^ ion.

## 1. Introduction

The rectifying (Schottky) metal–semiconductor (M-S) interface is an essential aspect of all electronic and photonic devices. Apart from its applications in electronic devices, the Schottky contacts are used as a tool for the study of semiconductors [1,2]. The transport of carriers across the M-S interface is influenced by the junction barrier potential. Therefore, it is of significant importance to study the current transport properties of the M-S interface and its modification for a better understanding of the operations of electronic devices [3,4].

3rd-generation semiconducting materials like gallium nitride (GaN), silicon carbide (SiC), and indium gallium nitride (InGaN), etc., have broad applications in power electronics, solid-state lighting, and microwave communication [5,6,7]. The electronic devices and photonic devices based on 3rd-generation semiconducting material like light-emitting diodes, photodetectors, diodes, solar cells, laser diodes, etc. have superior performance over 1st generation semiconducting material-silicon (Si) and 2nd-generation semiconducting material-gallium arsenide (GaAs) in terms of efficiency, frequency operation, temperature resistance, voltage resistance and radiation resistance etc. [5,6,8,9,10]. With invaluable material properties, GaN devices offer great potential for operation in wide temperature and pressure ranges and in strong radiation environments, which cannot be accomplished with customary semiconductor devices technologies currently available [11,12].

The M-S interface properties of the electronic devices are significantly altered in radiation-rich environments like space, high-altitude cusp regions, and nuclear reactors [13,14,15]. Ion irradiation is a method to simulate the effects of radiation rich, harsh environments on materials. MEI (range ~ 300 keV to 50 MeV) irradiation or implantation explores new applications in nanotechnology like modification in the properties of materials, devices and fusion of modern materials [13]. In MEI irradiation, both nuclear energy loss and electronic energy loss are comparable to each other and lead to combined effects or non-linear combined effects on damage production and damage recovery processes [15]. These effects of MEI irradiation lead to disorder build-up and microstructure expansion [16,17,18,19,20]. Studies on ion irradiation-induced effects in GaN semiconductor devices have been reported in the literature. Studies on GaN-based electronic devices irradiated by different SHIs have reported the formation of nano-tracks in GaN, development of nano-holes on the GaN surface, and an increase in resistivity of GaN [21,22,23,24]. The ion implantation of GaN by low energy ions reported the doping and amorphization of GaN [25,26]. Such damage not only induces micro-structural change, but also leads to a change in electrical properties. Most of the previous studies were performed on low-energy ion implantation and on the swift heavy ion (SHI) irradiation [21,22,27,28,29,30,31,32,33], but there have been only a few studies reporting on MEI irradiation [34,35]. Therefore, the investigation of the impacts of MEI irradiation on semiconductors and their device properties is important both from a fundamental and technological point of view.

In the present work, we selected 600 keV C^2+^ and 650 keV N^2+^ ions, whose ratio of nuclear energy loss (S_n_) to electronic energy loss (S_e_) is comparable. Stopping and Range of Ions in Matter (SRIM) is used to estimate the energy loss and Transport of Ions in Matter (TRIM) is used to generate the ionization and displacement damage profiles for MEI irradiation on Ni/Pd/n-GaN SBDs. Based on the estimations of the electrical parameters extracted, the current transport mechanism is explained, and the degree of damage is reported using Non-Ionizing Energy Loss (NIEL) and Linear Energy Transfer (LET). The present study reports 600 keV C^2+^ and 650 keV N^2+^ ion irradiation effects on Ni/Pd/n-GaN SBDs current transport properties for different fluences. Different models of extraction of electrical parameters of the Schottky interface are used to interpret the obtained results. X-ray diffraction (XRD) and photoluminescence (PL) studies are carried out to study the change in surface morphology and the formation of optically active defects by MEI irradiation.

## 2. Materials and Methods

The Schottky barrier diodes (SBDs) in the present study are fabricated on 2-µm-thick n(Si-doped)-GaN on the c-plane sapphire substrate. The detailed fabrication process of Ni/Pd/n-GaN SBDs can be found elsewhere [36]. To investigate the MEI irradiation effects on electrical properties of the Ni/Pd/n-GaN SBDs, the devices are exposed to 600 keV C^2+^ and 650 keV N^2+^ ion beams at room temperature. The beam current of MEI was 100 pnA. The ion fluences during the irradiation varied from 1 × 10^13^ to 1 × 10^15^ ions cm^−2^. The current-voltage (I-V) and capacitance-voltage (C-V) measurements were done using Semiconductor Device Parameter Analyzer (Agilent Technologies B1500A, Agilent, Santa Clara, CA, United States) as per Military Standard (MIL-STD) 750 E [37] at room temperature. Apart from this, X-ray diffraction (XRD, Malvern Panalytical Ltd., Malvern, United Kingdom) from 25 to 95 degrees is used to characterize the structural modifications at the Ni/Pd/n-GaN SBDs interface under MEI irradiation. The photoluminescence from wavelength 350 to 600 nm (PL-Fluorolog, Xenon lamp, 450 W, Excitation: 350 nm, resolution: 0.3 nm, Horiba, Kisshoin, Minami-ku Kyoto, Japan) were done to investigate the optically active interface defect states and their evolution during MEI irradiation.

## 3. Results

### 3.1. SRIM and TRIM Simulations

**Figure 1:**
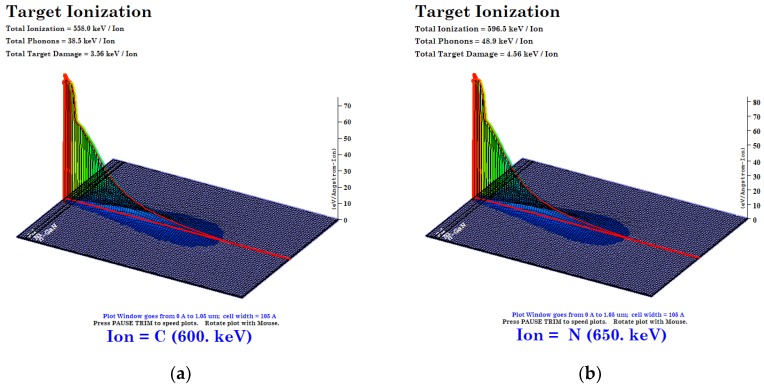

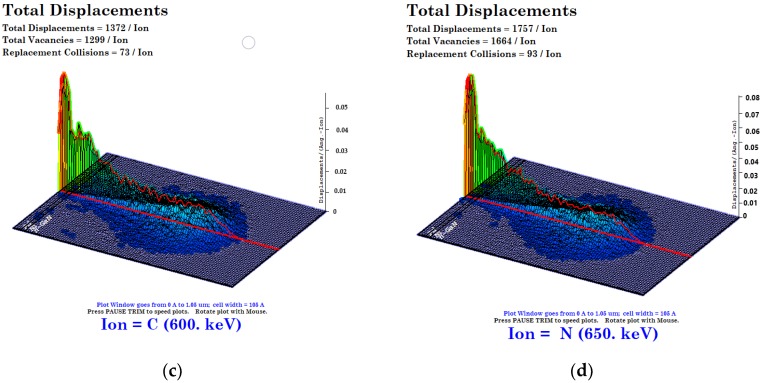
(**a**) Distribution of ionization losses for 600 keV carbon ions in Ni/Pd/n-GaN SBDs; (**b**) Distribution of ionization losses for 650 keV nitrogen ions in Ni/Pd/n-GaN SBDs; (**c**) Distribution of displacement losses for 600 keV carbon ions in Ni/Pd/n-GaN SBDs; (**d**) Distribution of displacement losses for 650 keV nitrogen ions in Ni/Pd/n-GaN SBDs.

The stopping range, non-ionizing energy loss (NIEL), linear energy transfer (LET) and ionization displacement profile was calculated (Table 1) for 600 keV C^2+^ and 650 keV N^2+^ ion irradiation using SRIM and TRIM simulations [38]. The procedure followed for the estimation of damage profile, LET, and NIEL is mentioned elsewhere [39,40]. From Figure 1a–d, it is evident that for both 600 keV C^2+^ and 650 keV N^2+^ ions, the ionization damage is dominant in the beginning and it diminishes consequently before the ions stop in the substrate. The particle traverses through the device structure and stops deep inside the substrate. This indicates that ionization and displacement damages are at their maximum at the M-S interface and decrease along with the depth of the semiconductor material. Hence, a large number of displacements/vacancies are generated due to displacement damage and electron-hole pairs, which are created due to ionization damage. The enormous amount of LET induces both ionization and displacement damage. Total ionizing dose (TID) is a function of LET, and hence in the case of 600 keV C^2+^ and 650 keV N^2+^ ions, the ratio of nuclear energy loss (S_n_) to electronic energy loss (S_e_) is found to be 3.1 × 10^−2^ and 3.2 × 10^−2^ respectively, which are comparable to each other. The fluence-dependent total ionizing dose (TID) and displacement damage (D_d_) are tabulated in Table 2.

### 3.2. Current-Voltage (I-V) Characteristics

**Figure 2:**
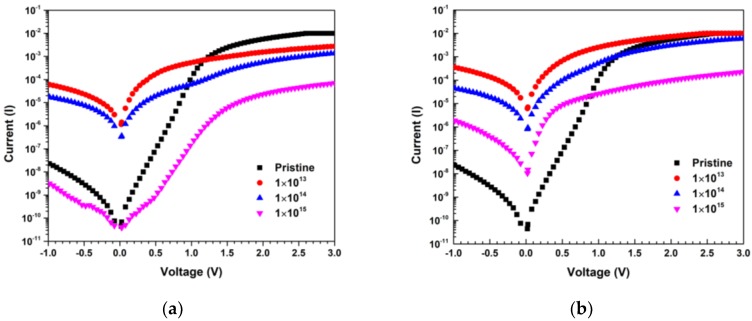
(**a**) I-V Characteristics of Ni/Pd/n-GaN SBDs for different fluences of 600 keV C^2+^ ions; (**b**) I-V Characteristics of Ni/Pd/n-GaN SBDs for different fluences of 650 keV N^2+^ ions.

Room temperature I-V characteristics under MEI irradiation for different fluences (1 × 10^13^ to 1 × 10^15^ ions cm^−2^) are shown in Figure 2a,b, respectively.

According to thermionic emission [41] theory, the rectifying metal-semiconductor (M-S) contacts show non-ideal I-V characteristics, given by

(1) $$I=I_{0}\exp\left(-\frac{q\left(V-IR_{S}\right)}{nkT}\right)$$

where the different symbols have their usual meanings [41].

For the diodes with large series resistance (R), Norde [42] developed a method to determine *φ_B_* and *R_S_*. Usually, the values of n lie between 1 and 2, so to determine large values of *R_S_* of diodes, the Norde’s method is used. Norde’s method involved the function *F(V)*, given as

(2) $$F\left(V\right)=\frac{V}{2}-\frac{kT}{q}\ln\left(\frac{I(V)}{{AA}^{*}T^{2}}\right)$$

The current *I*_0_ corresponding to minima of *F(V)* vs. *V* plot will give *R_S_* as

(3) $$R_{S}=\frac{\mathrm{kT}}{{\mathrm{qI}}_{0}}$$

and *φ_B_* is given as

(4) $$\varphi_{B}=F\left(V_{0}\right)+\frac{V_{0}}{2}-\frac{\mathrm{kT}}{q}$$

where *F(V_0_)* and *V*_0_ are the values of *F(V)* and *V* corresponding to the least value of current *I*_0_.

**Figure 3:**
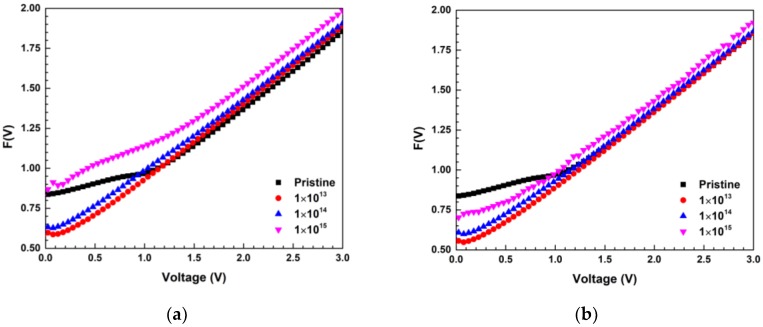
(**a**) The *F(V)* vs. *V* plot of Ni/Pd/n-GaN SBDs for different fluences of 600 keV C^2+^ ions; (**b**) *F(V)* vs. *V* plot of Ni/Pd/n-GaN SBDs for different fluences of 650 keV N^2+^ ions.

The extracted values of ideality factor (*n*), saturation current (*I*_0_) and barrier height (*φ_B_*) from *lnI* vs. *V* plot and series resistance (*R_S_*) and barrier height (*φ_B_*) values from Norde’s method [43] for different fluences of 600 keV C^2+^ and 650 keV N^2+^ ions from the graphs of *F(V)* vs. *V*. (Figure 3a,b) are reported in Table 3.

At the lower fluences, R_S_ decreases because of the increase in the carrier concentration due to the donor nature of carbon and nitrogen. Whereas at higher fluences, R_S_ increases due to the evolution of various defect levels at different positions in the band-gap [44,45]. When the incident ion comes to rest in the semiconductor, a damaged layer is produced along its trajectory, which leads to increased values of R_S_ [46]. The values of n are higher than unity for the pristine sample [36,47,48], which may be a result of the barrier inhomogeneities or due to tunneling and generation-recombination (G-R) currents, and it further increases with higher fluence due to increase in tunneling current. The reverse leakage current has increased significantly for the fluence of 1 × 10^13^ ions/cm^2^ of 600 keV C^2+^ and 650 keV N^2+^ ion irradiation. This increment in reverse leakage current occurs due to defects induced by 600 keV C^2+^ and 650 keV N^2+^ ion irradiation with deep energy levels in the middle of the forbidden gap which acts as generation-recombination (G-R) centers [49]. The displacements, vacancies introduced by 600 keV C^2+^ and 650 keV N^2+^ ion irradiation results in trap centers which leads to increase in thermal generation rate in the depletion region of the device. Thereafter there is a decrease in value of reverse leakage current along with the fluences confirms the increase of G-R centers [50]. The tunnelling of carriers through potential barriers by means of defect levels, i.e., defect assisted tunnelling significantly contributes to the increase in values of reverse leakage current. An increase in series resistance was found for higher irradiation fluences, demonstrating that the product of the mobility and carrier concentration has decreased. The decrease in mobility is due to the introduction of defect centers on irradiation, which act as scattering centers [40].

**Figure 4:**
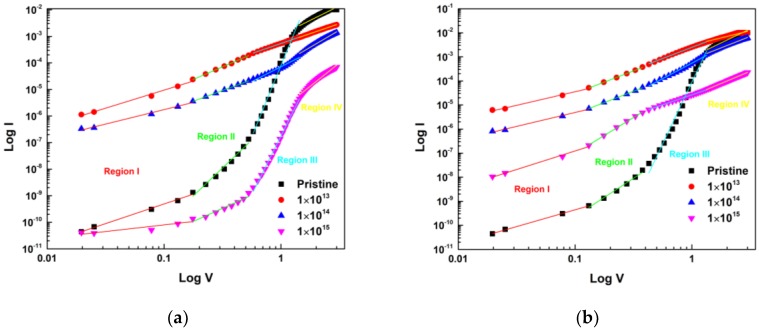
(**a**) The *logI* vs. *logV* plot of Ni/Pd/n-GaN SBDs for different fluences of 600 keV C^2+^ ions; (**b**) The *logI* vs. *logV* plot of Ni/Pd/n-GaN SBDs for different fluences of 650 keV N^2+^ ions.

The current passing through an interface can have contributions from different current transport mechanisms such as thermionic emission, tunneling and G-R mechanisms [51]. These individual contributions are dependent upon the nature of ions and their energy and fluence. To investigate the dominant current conduction mechanism of the Ni/Pd/n-GaN SBDs under 600 keV C^2+^ and 650 keV N^2+^ ion irradiation in the forward-bias region of I-V characteristics, *logI* vs. *logV* was plotted and are shown in Figure 4a,b [52]. The forward bias *logI* vs. *logV* plot of the Ni/Pd/n-GaN SBDs shows a power-law behavior of the current as

(5) $$I\;\propto \;V^{m}$$

where the exponent *m* values can be obtained from the slope of Figure 4a,b. The slope values *m*, indicate the different forward current conduction mechanisms of the device.

Here we can observe that *logI* vs. *logV* plot has four linear regions corresponding to low voltage region (I), intermediate voltage region (II and III), and high voltage region (IV) with different slopes given in Table 4, which shows the presence of various conduction mechanisms whereas the values of slope are found to be 1.48 (region I), 4.19 (region II), 10.76 (region III), and 2.18 (region IV) for the Ni/Pd/n-GaN SBDs, respectively. From the values of slope in region I, it is evident that the current conductions in the low bias region for the Ni/Pd/n-GaN SBDs exhibit an ohmic behavior because of existing background doping or thermally generated carriers [53]. In regions II and III, the slope estimations of the Ni/Pd/n-GaN SBDs are greater than two, indicating that the charge transport is governed by the trap-charge limited current (TCLC) due to the increase in the number of injected electrons which leads to filling up the traps [54]. At higher voltages (region IV), the values of slope tend to decrease as devices approach the “trap-filled limit”. This is a result of the strong electron injection; the electrons escape from the traps, which add to “space-charge-limited current (SCLC)” [55,56]. Obviously, at high voltages it reaches a trap-filled state and current conduction can be described by the trap-free Mott-Gurney law [57,58].

When the devices are exposed to 600 keV C^2+^ and 650 keV N^2+^ ion irradiation, the displacement and ionization damages are created along the trajectory of the ion beam, as is evident from the SRIM/TRIM simulations. This is reflected in the electrical characteristics of the device. For the low fluence of 600 keV C^2+^ and 650 keV N^2+^ ion irradiation, the slope of all four regions decreases, which may be due to the donor behavior of C^2+^ & N^2+^, which leads to filling up the traps and increasing the space charges. Whereas for the intermediate and highest fluences of 600 keV C^2+^ and 650 keV N^2+^ ion irradiation, the value of the slopes of regions II, III, and IV is continuously increasing, which may be due to the creation of defects, which act as traps, and charge transport is governed by the TCLC. The TCLC is more dominant for C^2+^ ions as compared to N^2+^ ions for the highest fluence, which may be due to the greater amount of damage caused by C^2+^ than N^2+^ ions in the device.

The reverse current conduction mechanism for 600 keV C^2+^ and 650 keV N^2+^ ion irradiation of Ni/Pd/n-GaN SBDs studied at room temperature by considering Poole–Frenkel emission (PFE) and Schottky emission (SE) mechanisms across the junction. The reverse current, when dominated by PFE mechanism, is given by [59]

(6) $$I_{R}\propto I_{0}\;\exp\left(\frac{\beta_{PF}V^{1/2}}{\mathrm{kT}d^{1/2}}\right)$$

and when the current is dominated by SE mechanism, it is given by

(7) $$I_{R}\propto \;AA^{*\;}\exp\left(-\frac{\varphi_{B}}{\mathrm{kT}}\right)\;\exp\left(\frac{\beta_{SE}V^{1/2}}{\mathrm{kT}d^{1/2}}\right)$$

where *β_PF_* and *β_SE_* are the PFE and SE field lowering coefficients, respectively. The theoretical values for *β_PF_* and *β_SE_* are given by

(8) $$\beta_{PF}=2\beta_{SE}={\left(\frac{q^{3}}{{\pi \varepsilon }_{0}\varepsilon_{r}}\right)}^{1/2}$$

The theoretical values of field lowering coefficients for Ni/Pd/n-GaN SBDs are *β_PF_* = 2.54 × 10^−5^ eVm^1/2^ V ^−1/2^ and *β_SE_* = 1.27 × 10^−5^ eVm^1/2^ V ^−1/2^.

**Figure 5:**
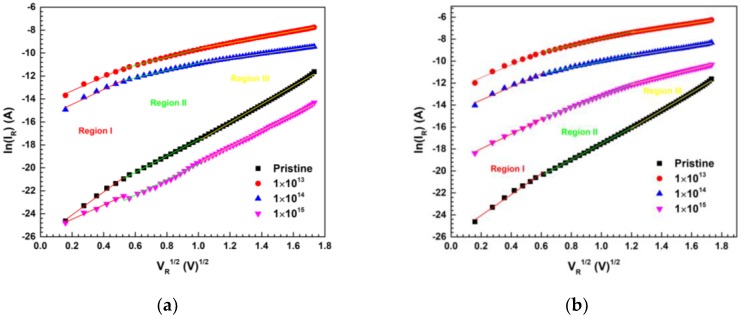
(**a**) The plots of *ln (I_R_)* vs. *V^1/2^* of Ni/Pd/n-GaN SBDs for different fluences of 600 keV C^2+^ ions; (**b**) The plots of *ln (I_R_)* vs. *V^1/2^* of Ni/Pd/n-GaN SBDs for different fluences of 650 keV N^2+^ ions.

From the plots of *ln (I_R_)* vs. *V^1/2^* (Figure 5a,b), the dominant reverse current transport mechanism is determined for MEI irradiation of Ni/Pd/n-GaN SBDs for different fluences at room temperature.

The results show (Table 5) that the reverse current conduction mechanism of Ni/Pd/n-GaN SBDs corresponds to PFE in the regions I, II, and III for pristine. This indicates that the carrier transport occurs from the metal into the conductive dislocation that occurred via a trapped state [60]. Then it changes to SE, which increases along with a fluence of 1 × 10^13^ ions cm^−2^ for irradiation with 600 keV C^2+^ and 650 keV N^2+^ ions due to the donor behavior of C^2+^/N^2+^ ions, which might have filled the trap states. Meanwhile, for higher fluences of C^2+^/N^2+^ ion irradiation, in region II and region III, the reverse current conduction mechanism of Ni/Pd/n-GaN SBDs corresponding to PFE may be due to the creation of defects which act as traps. Also, the PFE is more dominant for C^2+^ ions than for N^2+^ ions for the highest fluence, which may be due to the creation of a greater amount of damage by the C^2+^ ions than the N^2+^ ions in the Ni/Pd/n-GaN SBDs.

### 3.3. Capacitance-Voltage (C-V) Characteristics

**Figure 6:**
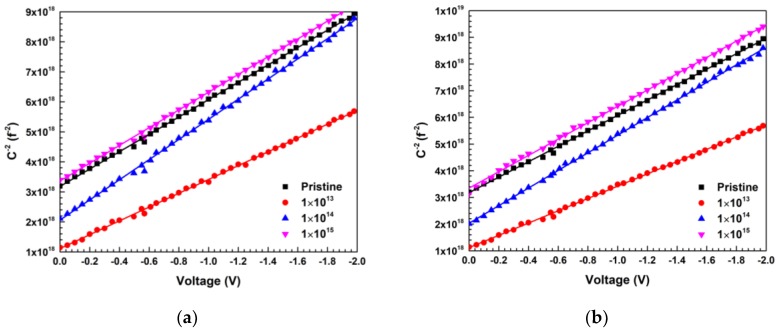
(**a**) The plots of *C*^−2^ vs. *V* of Ni/Pd/n-GaN SBDs for different fluences of 600 keV C^2+^ ions; (**b**) The plots of *C*^−2^ vs. *V* of Ni/Pd/n-GaN SBDs for different fluences of 650 keV N^2+^ ions.

The capacitance of MEI-irradiated Ni/Pd/n-GaN SBDs was measured as a function of junction voltage at a constant frequency of 1MHz. Figure 6a,b shows the variation of *C*^−2^ with the applied voltage of Ni/Pd/n-GaN SBDs, respectively, at room temperature for different fluences.

When a voltage V is applied to a junction, the capacitance of the depletion layer is given by [61]

(9) $$\frac{1}{C^{2}}=\frac{2}{A^{2}{q\varepsilon }_{0}\varepsilon_{r}N_{D}}\left(V_{i}-\frac{kT}{q}-V\right)$$

where the different symbols have their usual meanings [61].

The x-intercept (*V*_0_) of the *C*^−2^ vs. *V* plot is related to the built-in potential (*V_i_*)_,_ and the barrier height (*φ_B_*) is given by

(10) $$\varphi_{B}=\;V_{i}+\left(\frac{kT}{q}\right)ln\left(\frac{N_{C}}{N_{d}}\right)$$

The calculated values of *φ_B_* and dopant concentration (*N_d_*) are given in Table 6.

The values of *φ_B_* calculated by the I-V technique are smaller than the values extracted by the C-V technique. The presence of the native oxide (Ga_2_O_3_) layer at the M-S interface influences the I-V characteristics significantly [2,25]. All damage at the M-S interface changes the I-V characteristics since defects may act as recombination centers or traps for trap-assisted tunnel currents. Since the capacitance of the depletion layer is in series with the capacitance of the interfacial layer, the C-V measurements are less affected than I-V measurements. As I-V techniques involve the flow of electrons from semiconductor to metal, the barrier height extracted from this method will give lower value than from C-V measurements. This might be due to the image force lowering of the barrier height, whereas the value obtained from the capacitance measurement is not affected by image force [62]. In the present case, it might be because of the barrier inhomogeneities present at the M-S interface and the introduction of interfacial defects via displacement damage due to MEI irradiation [56,61]. The dopant concentration increases for the lowest fluence of 600 keV C^2+^ and 650 keV N^2+^ ions irradiation. From TRIM simulations, the ionization and displacement damage for 600 keV C^2+^ ions are 558.0 keV/ion, and 3.56 keV/ion, respectively, whereas for 650 keV N^2+^ ions their values are 596.0 keV/ion, and 4.56 keV/ion, respectively. Therefore, due to the lower value of *S_n_*, they cause less displacement damage as compared to ionization across the interface. This also leads to a significant decrease in the values of Schottky barrier height. Meanwhile, with higher fluence, the damage produced by avalanches will start overlapping, and several nontrivial effects may result. However, the increase in donor concentration at 1 × 10^13^ fluence is consistent with I-V data. The effective donor concentration may decrease with an increase in influence due to a possible increase in trap states due to defects. Here it is essential to take note that the I-V and C-V data are consistent with each other.

### 3.4. Photoluminescence (PL)

**Figure 7:**
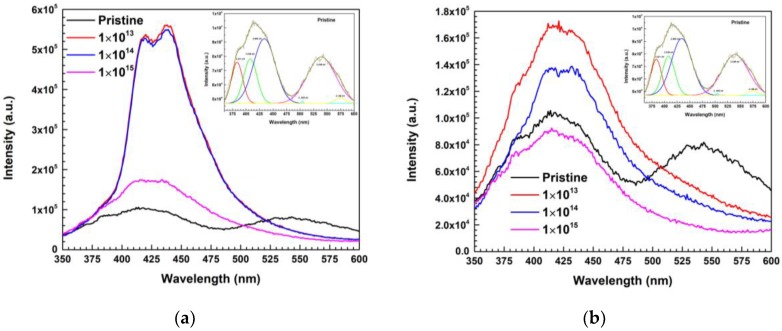
(**a**) The PL spectra of Ni/Pd/n-GaN SBDs for different fluences of 600 keV C^2+^ ions; (**b**) The PL spectra of Ni/Pd/n-GaN SBDs for different fluences of 650 keV N^2+^ ions.

The optically active interface defect states and their evolution during MEI irradiation were investigated based on PL characterizations performed at room temperature. PL spectra of 600 keV C^2+^ and 650 keV N^2+^ ions irradiated Ni/Pd/n-GaN SBDs are shown in Figure 7a,b.

The presence of Ga and N vacancies or deep-level impurities and amorphous phases in the pristine sample may be the reason for violet {VL (383.73 nm, 408.11 nm, 433.51 nm)}, cyan {CL (504.41 nm)}, green {GL (539.29 nm)} and yellow luminescence {YL (574.53 nm)} [9,63,64]. VL and CL increase for a fluence of 1 × 10^13^ ions cm^−2^ of 600 keV C^2+^ and 650 keV N^2+^ ion irradiation and steadily decreases with higher fluences, whereas YL vanishes in both the cases. Apart from that, BL (437.79 nm, 446.78 nm) evolved with a fluence of 1 × 10^13^ ions cm^−2^ of 600 keV C^2+^ and 650 keV N^2+^ ion irradiation and increased with average fluence, followed by a decline in the intensity of BL. Additionally, GL disappears for 1 × 10^15^ fluences of C^2+^ ion irradiation, whereas it decreases steadily with N^2+^ ion irradiation. This might be because of the donor behavior of C^2+^ ions and N^2+^ ions, which occupies the Ga vacancies and leads to the vanishing of YL and increases of VL, BL, CL, and GL at moderate fluences, and decreases afterward due to radiation damage [64,65,66,67]. The existence of GL luminescence may be expected due to the combination of gallium vacancy clusters and impurities (C^2+^/N^2+^) due to ion irradiation, which is bound to dislocations [67,68]. The results of PL are consistent with I-V and C-V data, reconfirming the observation that C/N are acting as donors at lower fluences.

### 3.5. X-Ray Diffraction (XRD)

**Figure 8:**
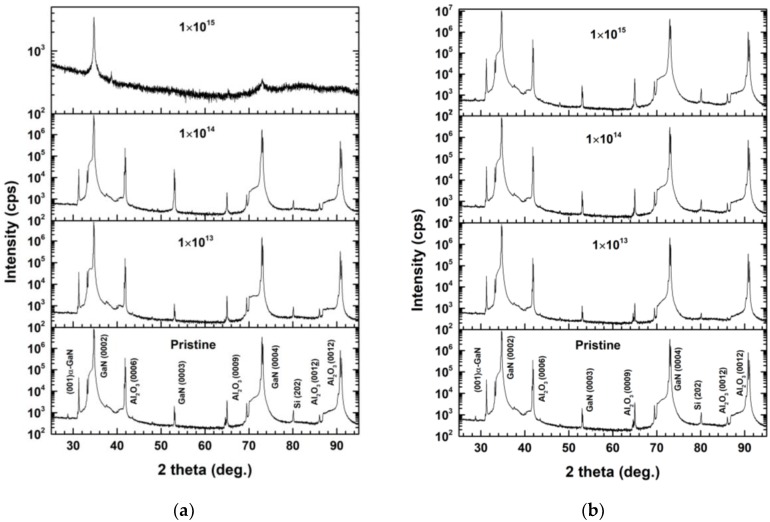
(**a**) The XRD pattern of Ni/Pd/n-GaN SBDs for different fluences of 600 keV C^2+^ ions; (**b**) The XRD pattern of Ni/Pd/n-GaN SBDs for different fluences of 650 keV N^2+^ ions.

The effect of MEI irradiation on the structural modifications at the Ni/Pd/n-GaN SBDs interface were investigated based on comparative XRD analysis, as shown in Figure 8a,b, covering from 25 to 95 degrees.

The results show that there are well-defined diffraction peaks of GaN (0002), Al_2_O_3_(0006), GaN (0003), Al_2_O_3_(0009), GaN (0004), Al_2_O_3_(0012), Al_2_O_3_(0012), Si (202) in the pristine sample [69,70,71]. MEI irradiation broadened the peaks of GaN (0002), Al_2_O_3_(0006) and GaN (0004). Also, there is no indication of any secondary phase formation in the MEI irradiated samples, which might be expected to be because of the high displacement damage introduced by ion irradiation [71,72]. The diffraction peaks are broadened, and a shift in the values of two thetas towards the lower side can be observed as the fluence of 600 keV C^2+^ and 650 keV N^2+^ ion irradiation is increased. Ion irradiation introduces lattice disorder into crystalline GaN, due to the expansion of which in the GaN peak, new peaks corresponding to the damaged section of the lattice evolved on the low theta side of the main GaN peak in the XRD spectra of the irradiated GaN [26,73,74]. Furthermore, it was observed that for higher fluences of C^2+^ ion irradiation, the dislocation density increased, leading to higher damage as compared to N^2+^ ion irradiation. The decrease in the intensity of C^2+^ ion irradiation is higher than that for N^2+^ ion irradiation, which may be due to the change in the mean atomic scattering factor at a given site for light C^2+^ ions as compared to N^2+^ ions [75,76]. Additionally, the peaks, except for GaN (0002), entirely disappear for the fluence of 1 × 10^15^ ions cm^−2^ of 600 keV C^2+^ ions, indicating that the GaN lattice is disordered, which might lead to the formation of a surface amorphous layer, whereas in the literature the formation of the surface amorphous layer has previously been reported for a fluence of 1 × 10^17^ ions cm^−2^ [77,78,79]. In the present case, ions are implanted in the device structure interstitially, leading to lattice deformations and the generation of defects. In the linear avalanche regime, the damage build-up might follow defect accumulation up to a critical defect density, and in the displacement avalanche regime, a disordered vicinity such as amorphization would arise because of direct ion impact mechanism [80]. Morehead and Crowder [81] proposed a model that hypothesized that each ion hitting the target produces a cylindrical amorphous core. Amorphization happens when such damage cores fill the area of the target. The critical fluence for amorphization decreases with increasing ion mass according to this semi-quantitative model. As nitrogen mass is higher than carbon, the amorphization is observed at a fluence of 1 × 10^15^ of C^2+^ ion, whereas there is no such type of observation for N^2+^ ion.

## 4. Discussion

The current transport properties of Ni/Pd/n-GaN SBDs were investigated for 600 keV C^2+^ and 650 keV N^2+^ ions in the fluence range of 1 × 10^13^ to 1 × 10^15^ ions cm^−2^ at room temperature. The electrical parameters such as ideality factor (*n*), series resistance (*R_S_*), and barrier height (*φ_B_*) were calculated using the I-V and C-V techniques. The increase in the value of *n* along with higher fluences of MEI irradiation may be due to the activation of multiple transport mechanisms. The values of *φ_B_* and *R_S_* decrease for a fluence of 1 × 10^13^ ions cm^−2^ and increase further for higher fluences of MEI irradiation. The contributions of various current transport mechanisms, including defect-assisted tunneling and G-R currents along with thermionic emission mechanisms might have significantly influenced the changes in values of *φ_B_* and *R_S_*. Usually, ion irradiation decreases the carrier mobility and carrier concentration and enhances the series resistance, which was also observed in the C-V analysis. MEI irradiation introduced a significant number of displacements and vacancies, which was also validated by the SRIM and TRIM calculations, and these defects increased the series resistance by decreasing the minority carrier [55]. Ionization and displacement damage profile simulations showed that the displacement damage was dominant in the bulk GaN. Additionally, 600 keV C^2+^ and 650 keV N^2+^ ions showed higher contributions of tunneling currents, as observed from the higher forward currents than reverse currents for fluences of 1 × 10^13^ to 1 × 10^15^ ions cm^−2^. The ratios of nuclear energy loss (S_n_) to electronic energy loss (S_e_) for 600 keV C^2+^ and 650 keV N^2+^ ions were comparable. This suggests that due to high nuclear energy loss, more displacement damage is created in the lattice, leading to massive contributions from defect-assisted tunneling currents [26].

Furthermore, the reverse current conduction mechanism of the Ni/Pd/n-GaN SBDs is PFE for lower reverse voltages and it changes to SE for higher fluences. Meanwhile, for higher reverse voltage, it was mainly SE and increased with higher fluences. The XRD analysis exhibited a broadening of peaks at higher fluences of 600 keV C^2+^ and 650 keV N^2+^ ions, which indicates the high degree of displacement damage created due to irradiation. At fluence 1 × 10^15^ ions cm^−2^ of 600 keV C^2+^ ions, the SBD showed an increase in ideality factor (*n*), and XRD showed structural degradation in Ni/Pd/n-GaN SBD, which were attributed to ion-induced damage in the form of point defects. Overall, C^2+^ and N^2+^ ion irradiation introduced similar damage in electrical characterization, whereas C^2+^ ion irradiation resulted in significantly higher structural damage compared to N^2+^ ion irradiation, which is evident from XRD. Additionally, PL characteristics of Ni/Pd/n-GaN SBDs for MEI irradiation indicate that at higher fluences yellow luminescence disappears, which is a signature of donor behavior of 600 keV C^2+^ and 650 keV N^2+^ ions, which occupies the Ga vacancies. Meanwhile, the green luminescence corresponds to the dislocation caused by MEI irradiation.

## 5. Conclusions

600 keV C^2+^ and 650 keV N^2+^ ion irradiation significantly altered the charge transport, structural and optical properties of Ni/Pd/n-GaN SBDs. The deviation in electrical parameters are correlated with the defects and the damage profiles estimated through SRIM/TRIM simulation, which are exceptionally reliant upon the fluences of MEI. Similarly, we affirmed that the charge transport mechanism is affected by additional defects at higher fluences due to the contributions of various other current transport mechanisms along with the thermionic emission mechanism. PL observation confirms that C/N act as donors at lower fluences, which is also validated by I-V and C-V measurements. From the XRD results, we conclude that structural damage in Ni/Pd/n-GaN SBDs is higher for 600 keV C^2+^ than 650 keV N^2+^ ions for a fluence of 1 × 10^15^ ions cm^−2^ which leads to the amorphization which is validated by semi-quantitative model.

## Figures and Tables

**Table 1:** TRIM Calculations in GaN SBDs.

Ion	Range, *R* (A^0^)	Displacement/Ion	Vacancies/Ion	Replacement Collisions/Ion	NIEL (MeV cm^2^/g)	LET (MeV cm^2^/g)	*S_n_/S_e_*
Carbon	6663	1372	1299	73	1.57 × 10^2^	1.47 × 10^3^	3.1 × 10^−2^
Nitrogen	6461	1757	1664	93	2.08 × 10^2^	1.90 × 10^3^	3.2 × 10^−2^

**Table 2:** Fluence dependent TID and D_d_ for GaN SBDs.

Fluence (Ions cm^−2^)	Carbon Ion		Nitrogen Ion	
	TID (Rad)	D_d_ (Rad)	TID (Rad)	D_d_ (Rad)
1 × 10^13^	2.36 × 10^8^	2.52 × 10^7^	3.03 × 10^8^	3.33 × 10^7^
1 × 10^14^	2.36 × 10^9^	2.52 × 10^8^	3.03 × 10^9^	3.33 × 10^8^
1 × 10^15^	2.36 × 10^10^	2.52 × 10^9^	3.03 × 10^10^	3.33 × 10^9^

**Table 3:** The values of *n*, *φ_B_*, *R_S_*_,_ and *I*_0_ from Rhoderic and Norde method and reverse leakage current of Ni/Pd/n-GaN SBDs for different fluences of 600 keV C^2+^ and 650 keV N^2+^ ions.

Ion	Fluence (Ions cm^−2^)	Ideality Factor (*n*)	Barrier Height (*φ_B_*) (eV)		Series Resistance (*R_S_*) (Ω)	Saturation Current (*I*_0_) (A)	Reverse Leakage Current at −1 V (A)
			*ln I* vs. *V*	*F(V)* vs. *V*			
Pristine		2.37	0.844	0.824	1.91 × 10^8^	6.41 × 10^−11^	2.73 × 10^−8^
Carbon	1 × 10^13^	2.76	0.582	0.598	4.56 × 10^3^	1.59 × 10^−6^	6.53 × 10^−5^
	1 × 10^14^	3.41	0.618	0.639	7.14 × 10^4^	3.85 × 10^−7^	1.87 × 10^−5^
	1 × 10^15^	4.34	0.860	0.856	6.68 × 10^8^	3.42 × 10^−11^	3.69 × 10^−9^
Nitrogen	1 × 10^13^	3.35	0.538	0.560	1.03 × 10^3^	3.85 × 10^−6^	3.75 × 10^−4^
	1 × 10^14^	3.29	0.591	0.611	7.49 × 10^3^	1.12 × 10^−6^	4.69 × 10^−5^
	1 × 10^15^	2.19	0.702	0.689	1.75 × 10^6^	3.85 × 10^−7^	2.15 × 10^−6^

**Table 4:** The slope from the *logI* vs. *logV* plot of Ni/Pd/n-GaN SBDs for different fluences of 600 keV C^2+^ and 650 keV N^2+^ ions.

	Fluence (Ions cm^−2^)	Slope Values			
Ion		Region I	Region II	Region III	Region IV
Pristine		1.48	4.19	10.76	2.18
Carbon	1 × 10^13^	1.36	1.89	1.59	1.50
	1 × 10^14^	1.09	1.62	2.23	2.56
	1 × 10^15^	0.90	1.98	8.84	3.15
Nitrogen	1 × 10^13^	1.27	1.86	1.85	1.19
	1 × 10^14^	1.15	1.89	2.57	1.91
	1 × 10^15^	1.54	2.98	6.65	2.03

**Table 5:** The experimental values of β_PF_ and β_SE_ of Ni/Pd/n-GaN SBDs are for different fluences of 600 keV C^2+^ and 650 keV N^2+^ ions.

Ion	Fluence (Ions cm^−2^)	Experimental Values (10^−5^ eVm^1/2^ V ^−1/2^)					
		Region I		Region II		Region III	
		β_PF_	β_SE_	β_PF_	β_SE_	β_PF_	β_SE_
Pristine		5.78	2.89	4.12	2.06	4.58	2.29
Carbon	1 × 10^13^	3.51	1.75	2.05	1.02	1.47	0.739
	1 × 10^14^	3.78	1.82	0.91	0.765	1.14	0.569
	1 × 10^15^	3.59	1.79	3.88	1.94	4.05	2.02
Nitrogen	1 × 10^13^	3.39	1.70	1.60	0.886	1.21	0.601
	1 × 10^14^	3.46	1.73	1.66	0.806	1.25	0.624
	1 × 10^15^	3.89	1.94	3.03	1.52	1.98	0.991

**Table 6:** The values of *φ_B_* and *N_d_* of Ni/Pd/n-GaN SBDs for different fluences of 600 keV C^2+^ and 650 keV N^2+^ ions.

Ion	Fluence (Ions cm^−2^)	*φ_B_* (eV)	*N_d_* (cm^−3^)
Pristine		1.146	3.38 × 10^17^
Carbon	1 × 10^13^	0.728	5.74 × 10^17^
	1 × 10^14^	0.799	3.45 × 10^17^
	1 × 10^15^	1.195	3.34 × 10^17^
Nitrogen	1 × 10^13^	0.677	5.58 × 10^17^
	1 × 10^14^	0.757	3.40 × 10^17^
	1 × 10^15^	1.063	3.29 × 10^17^
